# Reply regarding: Re-rupture is not the only failure: tendon elongation and the 6-week nonoperative protocol for Achilles tendon rupture

**DOI:** 10.2340/17453674.2026.46976

**Published:** 2026-10-19

**Authors:** Ville Happonen, Heikki Kröger

**Affiliations:** Department of Orthopaedics and Traumatology, Kuopio University Hospital and University of Eastern Finland, Kuopio, Finland

*Sir,*—We thank Spiezia and co-authors for their interest in our study [1] and for sharing their perspective on this important topic [2].

We agree that tendon elongation, muscle strength, return to activity, and patient-reported outcomes are important aspects of recovery after Achilles tendon rupture. As acknowledged in the limitations section of our manuscript, this data was unfortunately not available in this single-center cohort study from Finland.

We also agree that tendon healing and remodeling continue for several months after injury. In our treatment protocols, rehabilitation after immobilization was initiated cautiously, with gradual progression and the routine use of heel lifts for approximately 1–2 months after cast or orthosis removal.

The primary purpose of our study was to compare 2 nonoperative rehabilitation protocols rather than operative and nonoperative treatment strategies. The predefined primary endpoints were re-rupture and failure of nonoperative treatment requiring surgery, as these outcomes could be reliably identified from hospital records. Within the limits of this cohort and the available sample size, the observed rates of re-rupture and treatment failure requiring surgery were similar between the 6-week and 8-week protocols. Although tendon elongation may occur without leading to these endpoints, we are not aware of patients requiring subsequent surgical intervention because of tendon elongation after completion of nonoperative treatment.

Most operative procedures in our cohort were performed using open repair techniques, although a smaller number of minimally invasive procedures were also included. We agree that surgical techniques are relevant when interpreting complication rates and comparing operative and nonoperative treatment strategies.

Finally, we agree that future studies incorporating tendon length measurements, functional outcomes, return-to-activity data, and patient-reported outcome measures would provide valuable complementary information when evaluating rehabilitation protocols after Achilles tendon rupture.
